# The complete chloroplast genome sequence of *Heteropolygonatum ginfushanicum* (Asparagaceae) and phylogenetic analysis

**DOI:** 10.1080/23802359.2021.1933636

**Published:** 2021-05-31

**Authors:** Li Gu, Ting Su, Guang-Ling Luo, Guo-Xiong Hu

**Affiliations:** a College of Life Sciences, Guizhou University, Guiyang, China; b The Key Laboratory of Plant Resources Conservation and Germplasm Innovation in Mountainous Region Ministry of Education, Guizhou University, Guiyang, China; c Institute of Agro-bioengineering, Guizhou University, Guiyang, China

**Keywords:** Asparagaceae, *Heteropolygonatum*, plastome, polygonateae, nolinoideae

## Abstract

*Heteropolygonatum ginfushanicum* is an endemic epiphytic herb in China. The complete chloroplast (cp) genome of *H. ginfushanicum* is reported in this study. The total length of the cp genome is 155,508 bp with a typical quadripartite structure consisting of a large single copy region (LSC) of 84,552 bp and a small single copy region (SSC) of 18,528 bp, separated by a pair of 26,214 bp inverted repeats (IRs). It encodes a total of 113 unique genes, including 79 protein-coding, 30 tRNA, and four rRNA genes. Phylogenetic analysis indicated that *H. ginfushanicum* is sister to *Heteropolygonatum marmoratum* within subfamily Nolinoideae.

The genus *Heteropolygonatum* M. N. Tamura & Ogisu is a member of tribe Polygonateae of subfamily Nolinoideae in Asparagaceae (Tamura et al. 1997; Seberg et al. 2012). Species of *Heteropolygonatum* had been placed in the genus *Polygonatum* Mill. (Chao et al. 2013; Floden 2014a,b). Based on imbricate petals and the basic chromosome number of *x* = 16, Tamura et al. (1997) separated *Heteropolygonatum* from *Polygonatum*. Phylogenetic analyses also support this treatment, showing a sister relationship between the two genera (Xiao et al. 2017; Floden and Schilling 2018). *Heteropolygonatum* includes about 12 species and is mainly distributed in China and adjacent Vietnam (Tamura et al. 2000; Xiao et al. 2017; Floden 2018). Although four cp genomes of the genus have been reported, the plastome of *Heteropolygonatum ginfushanicum* was not involved and genome features of the genus are still unclear (Floden and Schilling 2018). In this study, the complete cp genome of *H. ginfushanicum* was sequenced to provide basic plastome features of *Heteropolygonatum*, which will contribute to systematics and phylogenetic study of the *Heteropolygonatum*.

The sample of *Heteropolygonatum ginfushanicum* was collected from Siping (107°34'48.42″E, 29°9'1.80″N, elevation 1464 m), Yangxi, Daozhen, Zunyi, Guizhou, China. Fresh leaves were put into silica gel to preserve until DNA extraction and the voucher specimens were deposited in the herbarium of the Natural Museum of Guizhou University (Voucher: Hu et al. 654, GACP). Total genomic DNA was extracted according to a modified CTAB method (Doyle and Doyle 1987). Paired-end (PE) reads of 150 bp was conducted on an Illumina Hiseq-2500 platform at BGI-Wuhan. Approximately, 2 GB raw data (13,676,984 Clean Reads) was generated and deposited in Sequence Read Archive (SRA) under accession number SRR13587437. Then paired-end reads of the clean data was filtered and assembled *de novo* using the GetOrganelle script with a mean coverage of 146 ×(Jin et al. 2020). The chloroplast genome was annotated using program PGA (Qu et al. 2019) with *Polygonatum odoratum* (NC_050926) (Du et al. 2020) as a reference, then coupled with manual adjustment using Geneious v.10.1.3 (Kearse et al. 2012). Analysis of boundaries between IRs and single copy regions was performed by online program IRSCOPE (Amiryousefi et al, 2018). Finally, the annotated complete cp genome of *H. ginfushanicum* was submitted to NCBI GenBank (Accession number: MW363694) and the circular genome map was generated with online program CHLOROPLOT (Zheng et al. 2020).

The complete cp genome of *Heteropolygonatum ginfushanicum* is 155,508 bp in length, and has a common quadripartite structure with a large single copy (LSC) of 84,552 bp and a small single copy (SSC) of 18,528 bp separated by a pair of inverted repeats (IRs) of 26,214 bp. The plastome of *H. ginfushanicum* is predicted to contain 113 unique genes, including a set of 79 protein-coding, 30 tRNA and four rRNA genes, of which 20 genes were duplicated in the IR regions. Among them, eight protein-coding genes (*atpF*, *ndhA*, *ndhB*, *petB*, *petD*, *rpl16*, *rpoC1*, *rpl2* and *rps16*) and five tRNA genes (*trnG^-UCC^*, *trnI^-GAU^*, *trnK^-UUU^*, *trnL^-UAA^* and *trnV^-UAC^*) contain one intron, and three genes (*clpP*, *rps12* and *ycf3*) include two introns. The overall GC content is 37.60%, while the corresponding value in the LSC, SSC, and IR regions is 35.61%, 31.40%, and 42.99%, respectively (Figure 1). As reported in other angiosperm (Mehmood et al. 2019, 2020a, b; Su et al. 2019, 2021), the IR regions have the highest GC content due to the presence of rRNAs containing high GC content. Analysis of boundaries between the IRs and single copy regions of *H. ginfushanicum* find that the *rps19* gene is 17 bp away from the LSC/IRb junction; The *ndhF* crosses the IRb/SSC boundary with a length of 29 bp in IRb and 2,182 bp in SSC and the *ycf1* gene spans the SSC/IRa boundary with a length of 890 bp in SSC which results in a pseudogene (*ψycf1*) at the IRa/SSC border (Figure 2).

**Figure 1.**
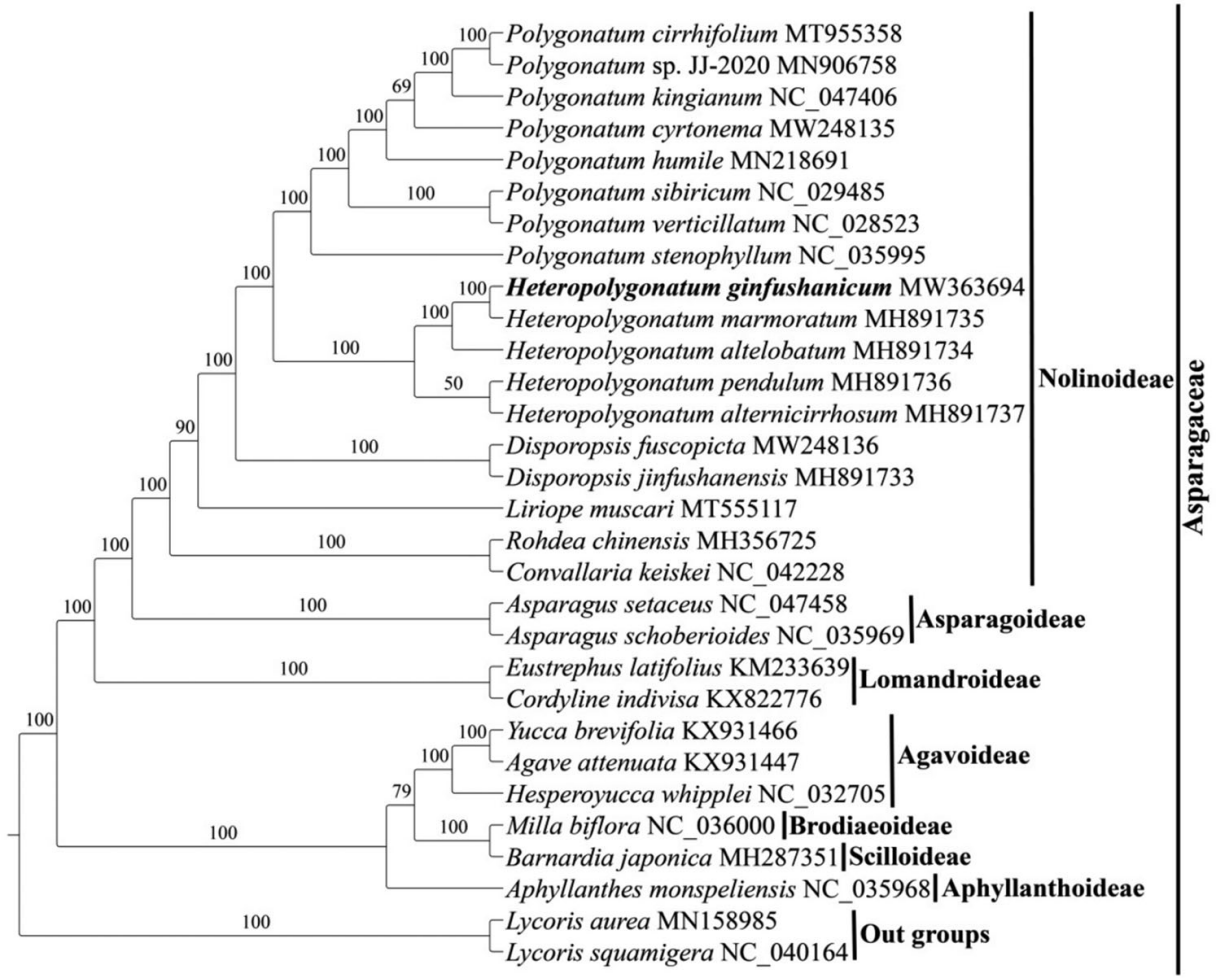
Chloroplast genome map of *Heteropolygonatum ginfushanicum*. The species name and specific information regarding the genome length, GC content are depicted in the center of the plot.

**Figure 2.**
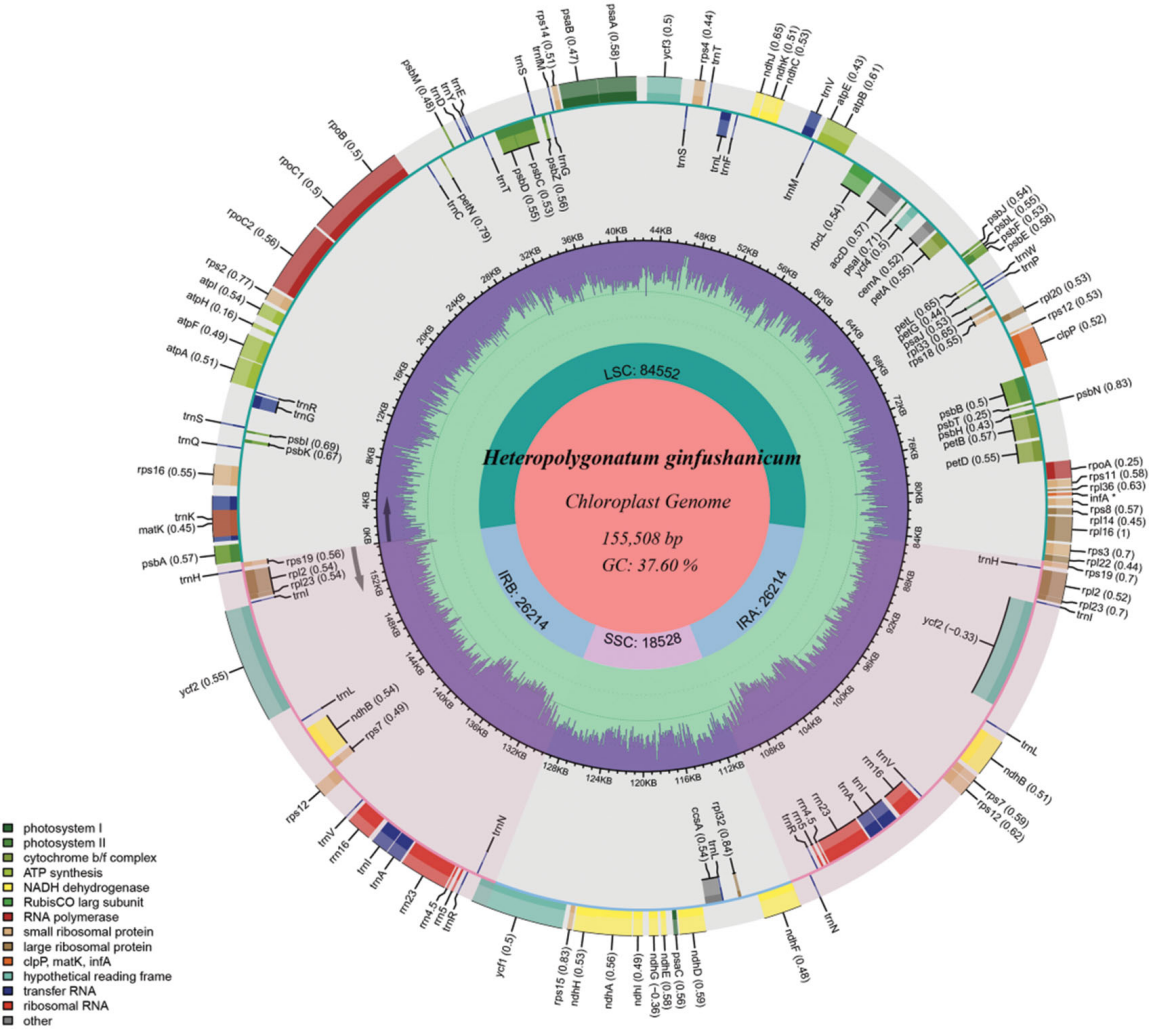
The boundary information between single copy and IR regions of chloroplast genomes of *Heteropolygonatum ginfushanicum*.

To explore the phylogenetic position of *Heteropolygonatum ginfushanicum* across the Asparagaceae, complete cp genomes of *H. ginfushanicum* and other 27 species of 7 subfamilies within Asparagaceae were selected to conduct analyses, using *Lycoris aurea* (MN158985) and *Lycoris squamigera* (NC_040164) from Amaryllidaceae as outgroups. Multiple sequence alignment of cp genome sequences were performed using MAFFT7.409 (Katoh and Standley 2013). Maximum likelihood (ML) analyses was conducted using RAxML-HPC2 on XSEDE v.8.2.12 (Stamatakis 2014) as implemented on the CIPRES Science Gateway (http://www.phylo.org/) (Miller et al. 2010) under the GTRGAMMA model. Bootstrap iterations (–#|–N) was set to 1000, and other parameters followed default settings.

Molecular phylogenetic analysis based on the cp genome sequences indicated that both *Polygonatum* and *Heteropolygonatum* are monophyletic and form a sister relationship within subfamily Nolinoideae, and *Heteropolygonatum ginfushanicum* is sister to *H. marmoratum* (Figure 3). This finding supports the separation of *Heteropolygonatum* as a distinct genus from *Polygonatum* (Meng et al. 2014; Xiao et al. 2017; Floden and Schilling 2018).

**Figure 3.**
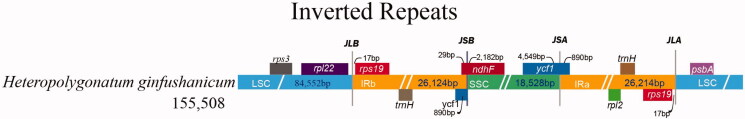
The maximum likelihood tree of Asparagaceae inferred from 30 complete chloroplast genomes with *Lycoris aurea* and *L. squamigera* (Amaryllidaceae) as outgroups. The position of *Heteropolygonatum ginfushanicum* is highlighted in bold.

## Supplementary Material

Supplemental Material

## Data Availability

The genome sequence data supporting this study are openly available in GenBank nucleotide database, https://www.ncbi.nlm.nih.gov/nuccore/MW363694, Associated BioProject, https://www.ncbi.nlm.nih.gov/bioproject/PRJNA698082, BioSample accession number at https://www.ncbi.nlm.nih.gov/biosample/ SAMN17705177 and Sequence Read Archive at https://www.ncbi.nlm.nih.gov/sra/ SRR13587437.
