# Supplementary figures and images for: The complete chloroplast genome sequence of Heteropolygonatum ginfushanicum (Asparagaceae) and phylogenetic analysis
Source: Mitochondrial DNA B Resour. 2021 May 31;6(7):1799–802. doi: 10.1080/23802359.2021.1933636 (PMC8168753; doi:10.1080/23802359.2021.1933636)

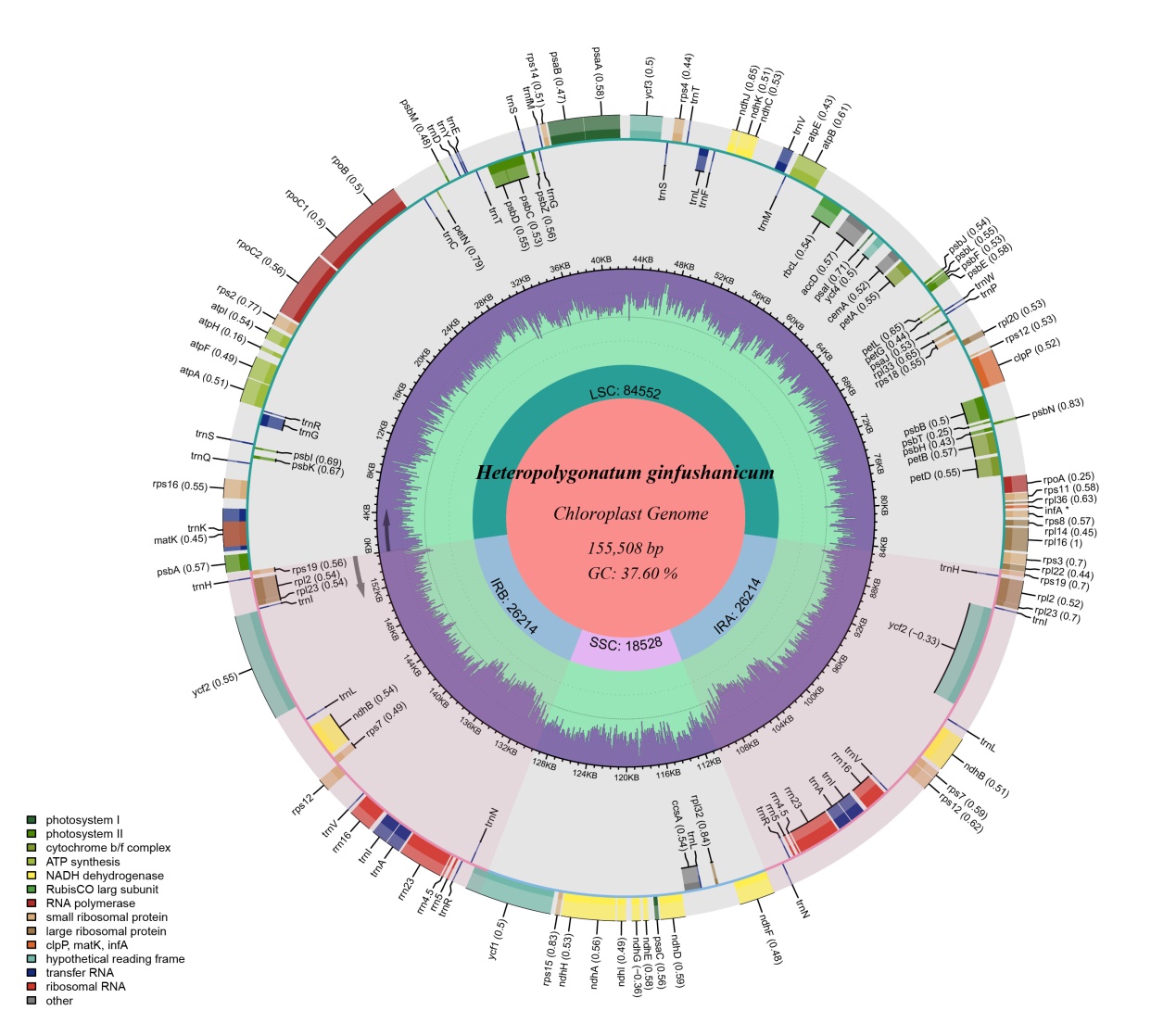


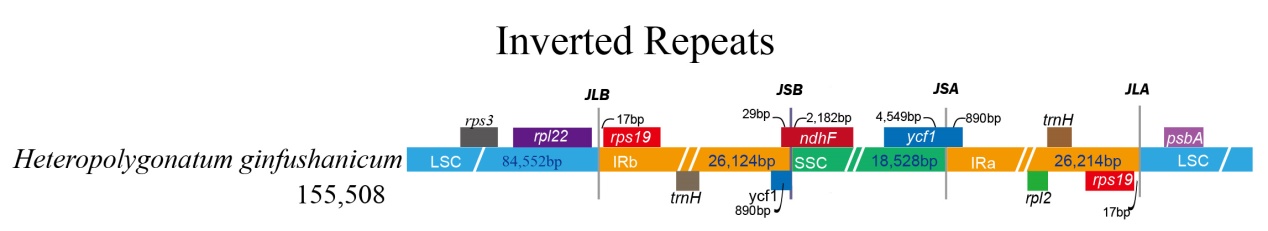

Supplement: Supplemental Material [file TMDN_A_1933636_SM3202.docx]
